# CDK8 as a Therapeutic Target for Overall Survival Prediction in Cervical Squamous Cell Carcinoma (CESC)

**DOI:** 10.2174/0115680096332050241022102545

**Published:** 2025-01-24

**Authors:** Feiting Xie, Qiugang Zhu, Shizhou Yang, Xiaofen Jin, Danqi Ruan, Lingfang Wang, Yang Li, Fang Ren, Xiaojing Chen

**Affiliations:** 1 Zhejiang Key Laboratory of Precision Diagnosis and Therapy for Major Gynecological Diseases, Women’s Hospital, Zhejiang University School of Medicine, China;; 2 Department of Laboratory Medicine, Shangyu People’s Hospital of Shaoxing, Shaoxing, China;; 3 Department of Gynecology and Oncology, Women’s Hospital, Zhejiang University School of Medicine, Hangzhou, Zhejiang, China;; 4 Zhejiang Provincial Clinical Research Center for Obstetrics and Gynecology, Hangzhou, Zhejiang, China;; 5 Zhejiang Key Laboratory of Maternal and Infant Health, Women’s Hospital, Zhejiang University School of Medicine, China;; 6 Department of Gynecology, First Affiliated Hospital of Zhengzhou University, No.1 Jianshe East Road, Zhengzhou, Henan, China

**Keywords:** CDK8, CESC, methylation, immune cell infiltration, diagnosis, prognosis

## Abstract

**Background:**

Cyclin-Dependent Kinase 8 (CDK8) is a paracrine transcriptional regulator involved in regulating cellular stress response, growth, and neurological functions in conjunction with mediator complex subunits 12 (MED12), MED13, and cyclin C.

**Objective:**

Studying the relationship between CDK8 and cervical squamous cell carcinoma (CESC) has significant clinical implications in diagnosis, treatment, and prognosis. Therefore, we analyzed the relationship between CDK8 and poor prognosis of CESC.

**Methods:**

Differentially expressed genes and their functional enrichment were analyzed using limma R software package. Immune cell infiltration was measured by TIMER and "GSVA" R packaging.

**Results:**

The results indicated that CDK8 was overexpressed in CESC, and the survival rate of patients in the CDK8 hypermethylation group was higher than that in the CDK8 hypomethylation group. In addition, CDK8 was associated with immune cell infiltration in tumor tissues.

**Conclusion:**

Overall, these findings provide more evidence for the relationship between CDK8 and a patients overall survival, which could provide insights into clinical diagnosis and prognosis prediction for CESC.

## 1 INTRODUCTION

Cervical Squamous Cell Carcinoma (CESC) is one of the most common gynecologic malignancies with a well-defined pathological staging, posing a major threat to women's health, especially in developing countries [1]. Among the tissue types of cervical cancer, about 75% to 80% are squamous cell carcinomas, 20% to 25% are adenocarcinomas, and a few are adenosquamous carcinomas, small cell carcinomas [2, 3]. Due to the lack of human papillomavirus (HPV) vaccination, early detection, and appropriate sex education, most patients progress to invasive stages of the disease [4, 5]. Surgery, chemoradiotherapy, and immunotherapy are currently the main options for CESC in therapy, but the mortality rate of cervical cancer remains high [6-10].

The occurrence and development of CESC are very complex biological processes, and despite extensive research, we still know very little about the basic molecular and pathogenic mechanisms of cervical squamous cell carcinoma [6, 11- 13]. Surgical resection and chemoradiotherapy cure most of the early patients, but for the late patients, they have little effect [14-21]. Once the recurrence and metastasis occur, patients are left with only a few treatment options [22-24]. Immunotherapy provides a new idea for clinical treatment [25-27]. Immune checkpoint inhibitors, tumor antigen-specific vaccines, and CAR-T cell therapy all bring new hope for patients to cure tumors [28, 29]. Several studies have also revealed the potential ability of PD-1/PD-L1, CTLA-4, CXCL8, and CXCL10 as targets for individuals to diagnose and predict prognosis [30-32]. In the future, the discovery of new targets for immunotherapies that reverse the suppressive immune environment and enhance the function of effector T cells may improve the cure rate of tumors [33-36]. Early screening and identification of specific therapeutic targets are also essential for prolonging disease-free survival in patients with CESC [37, 38]. Bulk RNA and DNA sequencing of tumor tissues support new perspectives to comprehend the mechanisms of the pathogenesis of CESC as well as to potentially establish a strong prognostic index and discover novel biomarkers, thereby improving the success rate of targeted therapy [39-41]. Recently, CDK8 has been found to play an important role in the cell cycle, tumorigenesis, metastasis, and prognosis [42, 43]. Nevertheless, its role in predicting cervical cancer prognosis has not been fully elucidated [44]. Cyclin-dependent kinase 8 (CDK8), a member of the CDKs known as a cell cycle regulator, has been identified as an oncogenic driver in several cancer types, including colorectal, breast, and prostate cancers [45-50]. CDK8 performs its physiological function through the formation of a kinase module with CCNC and mediator complex subunits (MED12 and MED13), exhibiting a dual role in different diseases or in different individual states of the same disease [51-53]. E966-05330-45418, an inhibitor of CDK, reduces prostate cancer migration and EMT by reducing CDK8 activity to inhibit TGF-β1-mediated Smad3/RNA pol II phosphorylation and Akt/GSK3β/ β-catenin signaling [45]. Senexin B, a CDK8/19 inhibitor, suppressed tumor growth and augmented the effects of fulvestrant in ER-positive breast cancer by inhibiting ER signaling [47]. In TNBC, CDK8 acts as a key regulator of NK cell-mediated immune escape, promoting primary tumor regeneration and metastatic spread [46, 54, 55]. Increasingly, studies have found that CDK8 expression is up-regulated in many types of cancers, such as CESC, gastric cancer, and colorectal cancer, and CDK8-deletion has implications for anti-tumor therapy [56-59]. Therefore, CDK8 can be used as a broad-spectrum tumor biomarker to develop CDK8-dependent novel treatment avenues [60].

To determine whether CDK8 could serve as a biomarker for early diagnosis and prognostic evaluation, we comprehensively explored its expression and its prognostic significance in CESC. We then discussed the effects of CDK8 on prognosis, clinical features, methylation, immune cell infiltration, and biological function. Finally, we validated CDK8 expression in clinical samples and its partial biological function *in vitro*.

## 2 MATERIALS AND METHODS

### 2.1 Experimental Methods

#### 2.1.1 Cell Culture and Transfection

The cell lines, SiHa and HeLa, from the American Type Culture Collection (ATCC) were cultured in DMEM supplemented with 10% FBS and antibiotics (1% penicillin-streptomycin) at 37 °C under 5% CO_2_. Specific siRNAs targeting CDK8 were obtained from Tsingke Biotechnology Co., Ltd. (Beijing, China). Afterward, 1.2 mL mixed liquids (1 mL DMEM with 2% FBS, 192 μL GenMute™ Transfection Buffer (SL100572, SignaGen) in working solution, 3 μL GenMute™ (SL100568, SignaGen), and 5 μL siRNAs were transfected into SiHa and HeLa cells to down-regulate the expression of CDK8.

#### 2.1.2 Western Blot Analysis

Proteins were extracted from whole-cell lysates. After electrophoresis on 4%-20% SDS–PAGE gels, proteins were transferred onto PVD. The rabbit anti-CDK8 (1:2000; Proteintech) and mouse anti-β-actin (1:5000; Proteintech) were used.

#### 2.1.3 Cell Counting Kit-8

Cells were evenly seeded into the 96-well plates at a density of 5000 cells/well and allowed to incubate for 24 h, 48h, 72h, and 96h. Cell viability was measured using the cell counting kit-8 (A311-01, Vazyme) at various time points.

### 2.2 Colony Formation Assay

The cells were inoculated into 12-well plates for 1-2 weeks, with periodic changes in the complete medium. When there were visibly cloned colonies in the 12-well plate with approximately 50-150 cells per clone, the culture was terminated. The 12-well plate was washed with PBS twice, and then 800 μL of 4% paraformaldehyde was added and incubated at room temperature for 20 min to fix the cells. The plate was then washed with PBS again and stained with 800 μL of 0.2% crystal violet solution for 20 min. The plate was then thoroughly washed with deionized water and allowed to air dry before being photographed and analyzed for cell counting and statistical analysis.

#### 2.2.1 RNA Extraction, cDNA Synthesis, and qRT-PCR

Seven paired CESC and adjacent normal tissues were collected. Total RNA extraction was performed using Trizol reagent (15596026, Thermo Fisher, USA), followed by cDNA synthesis using the RT-PCR kit (HF001-01, Vazyme, Nanjing). Quantitative real-time PCR (qRT-PCR) was conducted using HiScript II Q RT SuperMix (R223-01, Vazyme, Nanjing), as previously described.

#### 2.2.2 Apoptosis Analysis

Apoptosis was detected by flow cytometry using the Annexin-V /PI apoptosis detection kit according to the manufacturer's instructions.

### 2.3 Bioinformatic Analyses

#### 2.3.1 Obtaining the Expression Profiles and Clinical Data of CESC

The expression and clinical data of patients were downloaded from the TCGA database (https://portal.gdc.cancer.gov/). The high-throughput sequencing dataset GSE6791 was downloaded through the GEO database (https://www.ncbi.nlm.nih.gov/geo), including 20 cervical cancer tissues and 8 normal cervical epithelial tissues.

#### 2.3.2 Functional Enrichment Analyses

Correlated genes of CDK8 were identified using the online database GeneMINIA (http://genemania.org/). Functional enrichment analysis was conducted using Metascape (https://metascape.org/gp/index.html#/main/step1).

#### 2.3.3 Identification of Differentially Expressed Genes (DEGs)

Differentially expressed genes (DEGs) between two groups were identified using the 'Limma' R package. The cutoff criteria were set as follows: |logFC| > 0.25 and Adj.P-Value < 0.05. Volcano plots depicting these results were generated using the 'ggplot2' R package [60].

#### 2.3.4 Gene Mutation Analysis

The mutations of CDK8 in CESC patients were explored using the online database Gene Set Cancer Analysis (GSCA) (http://bioinfo.life.hust.edu.cn/GSCA/#/) [60].

#### 2.3.5 Measurement of Immunocyte Infiltration

The ssGSEA algorithm was employed to measure the infiltration score of 24 common immunocytes using the “GSVA” R package. The relationships between risk score and immunocyte infiltration were assessed using Spearman correlation coefficients [60].

### 2.4 Statistical Analysis

The data was analyzed by R software. Kaplan-Meier plotter analysis was carried out to assess the impact of CDK8 on patients' prognosis. Univariate and multivariate Cox regression analyses were conducted to evaluate the effects of CDK8 on patients' prognosis. Spearman correlation analysis was utilized to examine the relationship between risk score and immunocyte infiltration. Statistical significance was defined as follows: ns: not significant; *p < 0.05; **p < 0.01; ***p < 0.001; ****p < 0.0001, ns not significant.

## 3 RESULTS

### 3.1 Functional Enrichment Analysis

Firstly, we used the Spearman test to analyze the expression of CDK8 in CESC (Fig. **1A**). To further explore the differences between positively correlated genes and negatively correlated genes of CDK8, we identified the DEGs between positively and negatively genes array (Figs. **1B** and **C**). Functional enrichment analyses revealed signaling pathways and cellular activities in which CDK8 is primarily involved (Figs. **1D** and **1E**).

### 3.2 CDK8 is Highly Expressed in CESC

Pan-cancer analysis showed that the expression of CDK8 was upregulated and elevated in tumor tissues compared to normal tissues in CESC (Figs. **2A-D**).

### 3.3 Analysis of Methylation and Gene Copy Number of CDK8 in CESC

A methylation study was performed on CDK8 using the TCGA cohort. Correlation analysis showed that CDK8 methylation level was negatively correlated with mRNA expression in CESC (Fig. **3A**). Survival analysis revealed that hypermethylation of CDK8 was associated with better overall survival (OS) for CESC patients (Fig. **3B**). Analysis of the 15 methylated sites on CDK8 led to the conclusion that the methylation was negatively correlated with CDK8 expression (Fig. **3C**).

### 3.4 CNV and SNV analysis of CDK8 in CESC

We initially used GSCA to explore the gene mutation types of CDK8 in CESC. The mutation types of CNV in CESC and their corresponding proportions are shown in the pie charts (Fig. **4A**). Spearman correlation analyses revealed a positive correlation between CDK8 CNV and mRNA expr- ession in CESC (Fig. **4B**). Kaplan-Meier survival analyses showed overall survival (OS) (*p* = 0.01) and progression-free survival (PFS) (*p* = 0) in patients with wild-type CDK8 compared to those with copy-number amplification or deletion (Figs. **4C** and **D**). The mutation types were mainly missense substitution (50%) and synonymous substitution (50%) in the C/G bases (C>T (25%), C>G (25%), G>A (50%)) Figs. **4E** and **F**).

### 3.5 Relationships between CDK8 and Immune Cell Infiltration in CESC

Based on the above findings, we further explored the effect of CDK8 on immune cell infiltration in CESC [60]. The risk score showed a positive correlation with Tcm, T helper cells, Th2 cells, Tgd, and eosinophils in cohorts (Fig. **5A**). The result of the immune score was consistent with the above analysis that CDK8 was negatively linked to the immune infiltration (Fig. **5B**).

### 3.6 Verification of CDK8 in CESC Samples and Cell Lines In vitro

Finally, we detected the expression of CDK8 in cervical cancer tissues and paracancerous tissues by qRT-PCR and western blotting. As shown in Figs. (**6A**-**B**), CDK8 was significantly upregulated in cervical cancer tissues. This result was basically consistent with the findings of the TCGA cohort. The down-regulation of CDK8 significantly reduced the proliferation and colony-forming ability of Hela and SiHa cells but had no significant effect on apoptosis (Figs. **6C**-**H**). All these findings confirmed that CDK8 plays a critical role in CESC.

## 4 DISCUSSION

Cervical cancer is now the fourth most common cancer worldwide, with cervical squamous cell carcinoma (CESC) accounting for approximately 75-80% of all cervical cancer tissue types [61, 62]. There has been a long-term decline in cervical cancer incidence and mortality worldwide since universal cervical cancer screening and human papillomavirus vaccines [63-65]. However, the mortality rate of cervical cancer in China has not been reduced to the expected level, and the 5-year survival rate of metastatic cervical cancer patients is only 16.5% [66]. Therefore, the pathogenesis, potential therapeutic targets, and early diagnostic markers of cervical cancer deserve more in-depth exploration. With the rapid development of high-throughput detection and analysis technologies, the integrated use of bioinformatics databases and corresponding methods contributes to the discovery of novel tumor diagnostic markers and also provides new insights into cancer diagnosis [6]. Through the analysis of the bioinformatics database, we found that the expression of CDK8 in CESC was higher than that in normal cervical epithelial tissues. Further functional enrichment analyses revealed the signaling pathways and the cellular activities in which CDK8 is primarily involved. Subsequent analyses revealed that the mutation type and methylation level of CDK8 were significantly correlated with clinical parameters associated with the patients. The expression of CDK8 was negatively correlated with immune cell infiltration. Ultimately, we validated the expression levels of CDK8 in CESC samples and its biological functions in cell lines.

CDK8, a member of the human cyclin-dependent kinase family, exerts its pro-oncogenic functions by binding to regulatory partners [48, 67, 68]. Depending on the environmental stimuli and cell type, the function of CDK8 can appear to be diverse, which seems to explain the multiple possibilities of CDK8 in transcriptional regulation [69]. As a product of proto-oncogenes, CDK8 is involved in cell cycle regulation, cell division, and other processes, and its overexpression can accelerate cancer cell growth and division process [29, 47]. Here, we first confirmed from different datasets that high expression of CDK8 excels in predicting poor clinical prognosis in CESC. In our study, we explored for the first time the relationship between the methylation level of CDK8 and the prognosis of CESC patients. Prognostic analyses revealed that patients with CDK8 hypermethylation tended to have better OS. Secondly, we briefly described the negative relationship between CDK8 and immune cell infiltration.

Upregulation of CDK8 expression has been found in a variety of tumors, and it is related to tumor invasion, metastasis, prognosis, and chemotherapy resistance [40, 70]. All of the above findings may suggest that CDK8 may be a new therapeutic target and marker of clinical prognosis for CESC. Interestingly, CDK8 is also highly expressed in immune cells, inhibiting anti-tumor surveillance and causing immune escape of tumors. The application of CDK8 small molecule kinase inhibitors can not only inhibit the tumor but also enhance the function of immune cells to a certain extent. Therefore, this method is also effective in antiviral immunity [71, 72]. T cells are a major component of the immune system, which is responsible for controlling and regressing cancer. Based on the above conclusions, we may conduct genome-wide CRISPR knock-out in human-engineered T cells in the future and then screen out more specific activation and killing abilities [26]. Studies have reported that the loss of CDK8 reverses the immune evasion process of primary tumor recognition mediated by NK cells [73]. CDK8 can regulate the differentiation of Tregs, which makes it possible for the adoptive transfer of Tregs to treat pathological inflammation or autoimmune [74, 75]. Moreover, CDK8 is correlated with the expression of PD-L1 in tumors, which provides a theoretical basis for the combination of CDK8 small molecule inhibitors and immune checkpoint inhibitors in the future [46, 76]. In the next five years, we expect that CDK8 will continue to be explored as a key molecular target in CESC. Advances in immunotherapy, coupled with a deeper understanding of the role of CDK8 in immune cell infiltration and tumor progression, may lead to the development of novel therapeutic strategies that improve patient outcomes.

Overall, our study provides new insights into the regulation of the immune microenvironment and immunotherapeutic treatment of CESC, but our study still has some limitations. Firstly, in view of the difficulty of paired clinical samples and the long collection period, our experimental design is relatively simple. Secondly, the mechanisms by which CDK8 contributes to immune evasion and its interactions with other signaling pathways in CESC require further exploration. Thirdly, further studies are needed to validate the relationship between CDK8 hypermethylation and improved patient survival in larger, more diverse cohorts. Last, more evidence needs to be collected, including clinical samples, and further *ex vivo* and *in vivo* experiments should be conducted to validate the function of CDK8.

## CONCLUSION

In conclusion, we validated the expression and prognostic significance of CDK8, providing new insights into predicting the prognosis and clinical guidelines for individualized treatment of CESC. The potential effects and mechanisms of CDK8 on methylation and immune cell infiltration in CESC require further exploration in future studies.

## Figures and Tables

**Fig. (1):**
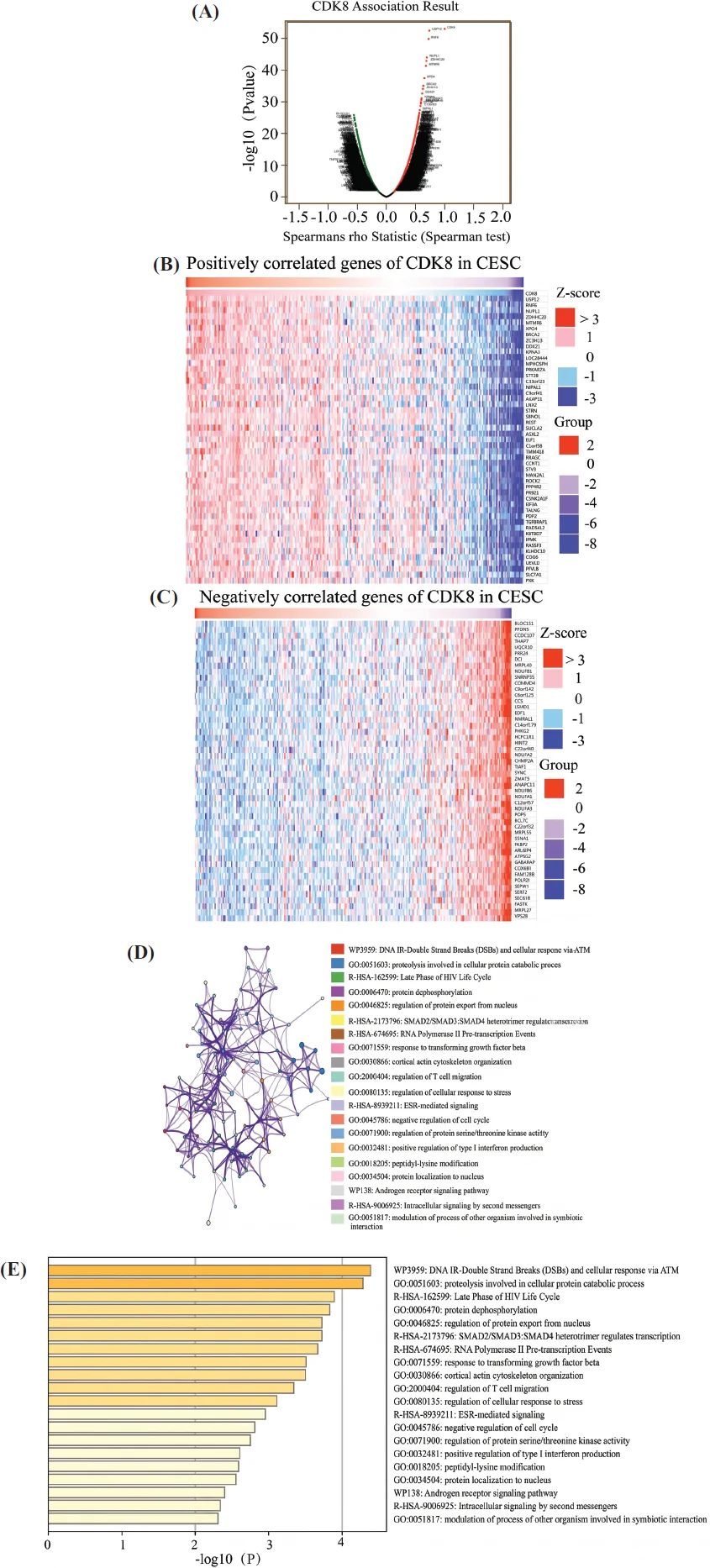
Functional enrichment analysis of CDK8. (**A**) The expression of CDK8 in CESC. (**B**) and (**C**) Positively and negatively correlated genes of CDK8 in CESC. (**D**) and (**E**) Functional enrichment analysis of CDK8 in CESC.

**Fig. (2):**
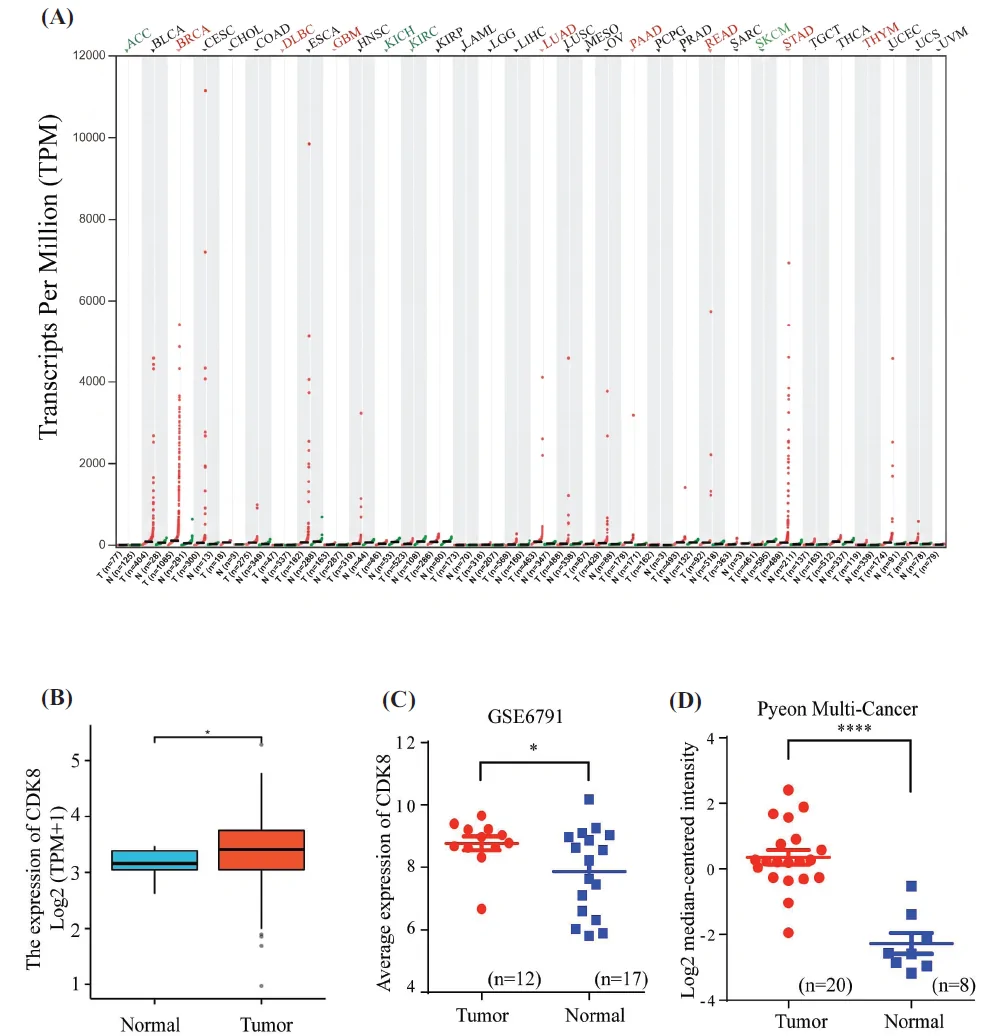
The expression levels of CDK8 in CESC. (**A**-**D**) The relative expression levels of CDK8 in tumor and normal tissues.

**Fig. (3):**
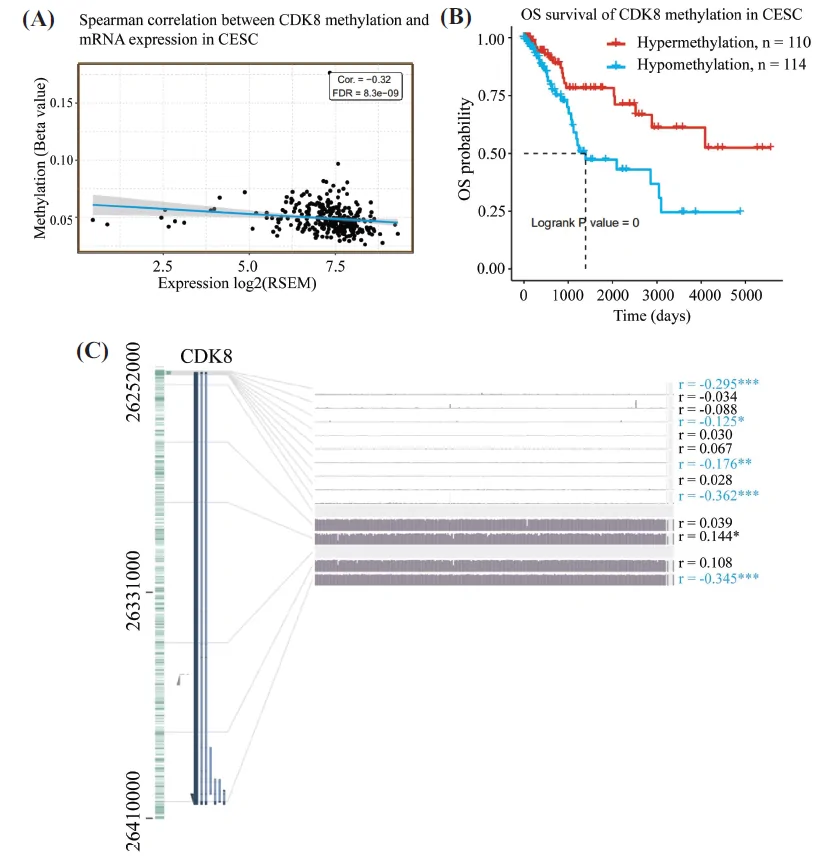
Relationship between CDK8 methylation and overall survival in CESC. (**A**) Spearman correlation between CDK8 methylation and mRNA expression in CESC. (**B**) The relationship between CDK8 methylation and OS in CESC. (**C**) Fifteen methylated sites on CDK8 in CESC.

**Fig. (4):**
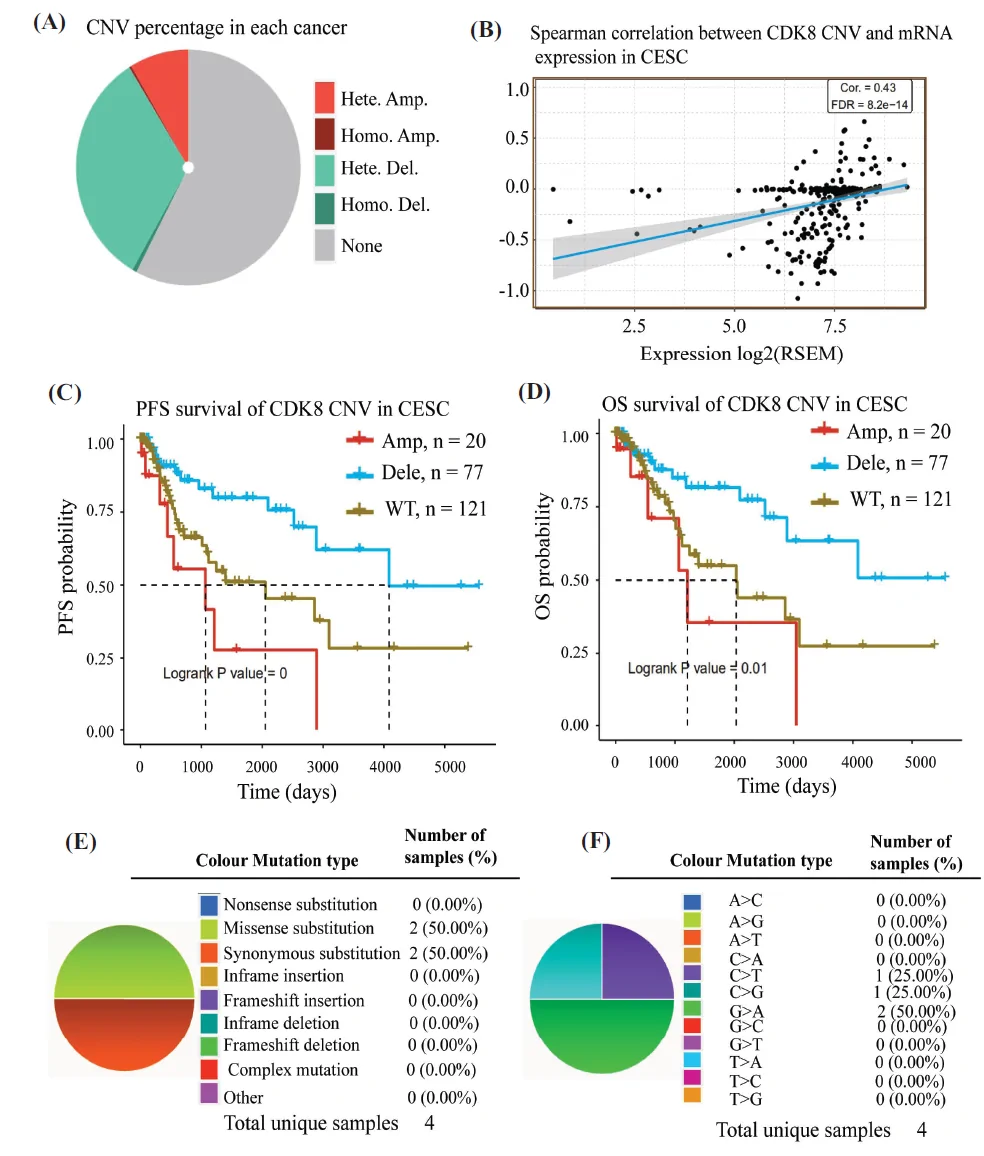
Gene mutation analysis of CDK8 in CESC. (**A**) The mutation types of CDK8 regarding CNV in CESC. (**B**) Correlation analyses between CNV and mRNA expression. (**C**) The effects of gene set CNV on prognosis in CESC. (**D**) The mutation types and their corresponding proportions of CDK8 regarding SNV in CESC.

**Fig. (5):**
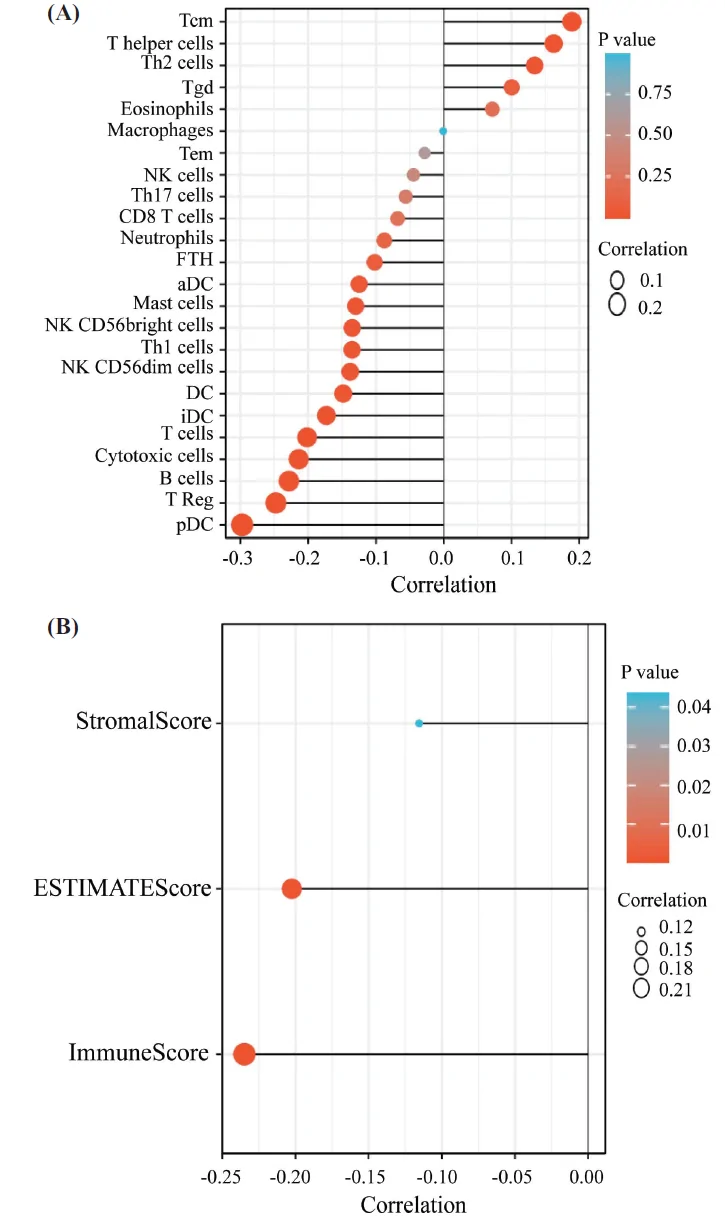
The relationships between the expression of CDK8 and immunocyte infiltration in CESC. (**A**) and (**B**) Different immunocyte infiltration in CESC.

**Fig. (6):**
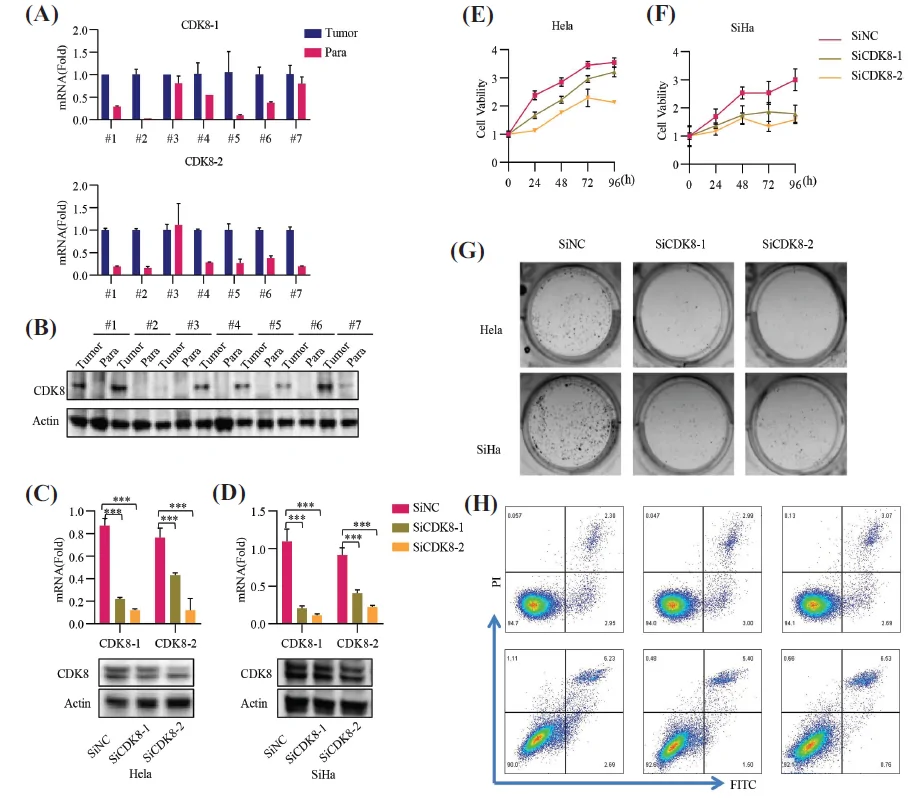
Functional verification of CDK8 by experimental methods *in vitro*. (**A**) and (**B**) Detected the expression of CDK8 in cervical cancer tissues and para-carcinoma tissue by qRT-PCR and western blotting. (**C**) and (**D**) Detection of knockdown efficiency of different RNAs in Hela and SiHa by qRT-PCR and western blotting. (**E**) and (**F**) The changes in cell proliferation in Hela and SiHa after CDK8 was knocked down. (**H**) The changes in colony formation in Hela and SiHa after CDK8 was knocked down. (**G**) The changes in apoptosis in Hela and SiHa after CDK8 was knocked down.

## Data Availability

The datasets used and/or analyzed during the current study are available from the corresponding author upon reasonable request.

## LIST OF ABBREVIATIONS

CCNC: Cyclin C
CDK8: Cyclin-dependent kinase 8
CESC: Cervical Squamous Cell Carcinoma
HPV: Human Papillomavirus
MED12: Mediator Complex Subunit 12
MED13: Mediator Complex Subunit 13
OS: Overall Survival
qRT-PCR: Quantitative Real-Time Polymerase Chain Reaction
siRNA: Small Interfering RNAs
